# Exogenous choline chloride enhances salt tolerance in wheat and its underlying physiological mechanisms

**DOI:** 10.3389/fpls.2025.1671734

**Published:** 2025-09-24

**Authors:** Feng Zhou, Guoquan Wang, Panpan Lu, Yan Liu, Zengbing Guo, Xinhui Wang, Zifan Zhou, Li Xu, Ying Zhang, Weiguo Li, Runqiang Liu

**Affiliations:** ^1^ School of Plant Protection and Environment, Henan Institute of Science and Technology, Xinxiang, China; ^2^ Henan Engineering Research Center of Green Pesticide Creation and Pesticide Residue Monitoring by Intelligent Sensor, Henan Institute of Science and Technology, Xinxiang, China

**Keywords:** choline chloride, salt stress, wheat, growth characteristics, physiological mechanism

## Abstract

Soil salinity is a major abiotic stress that seriously impairs crop growth and development, limiting global food production. As a primary staple food, wheat reduced grain yield and quality under salt stress, posing significant challenges to food security. Recent studies indicate that choline chloride, a safe and efficient plant growth regulator, can alleviate drought symptoms in wheat seedling and enhance crop salt tolerance.In this study, the growth and physiological indexes of wheat seedlings were determined by hydroponics.The current study demonstrates that application of 400 mg.L^-1^ choline chloride effectively mitigates salt stress symptoms in wheat seedlings. Specifically, it increases leaf chlorophyll content while reducing osmic and oxidative stress biomarkers. Furthermore, choline chloride treatment significantly boosts the activity of key reactive oxygen species (ROS)-scavenging enzymes. These findings hold considerable promise for famers cultivating saline soils.

## Graphical Abstract

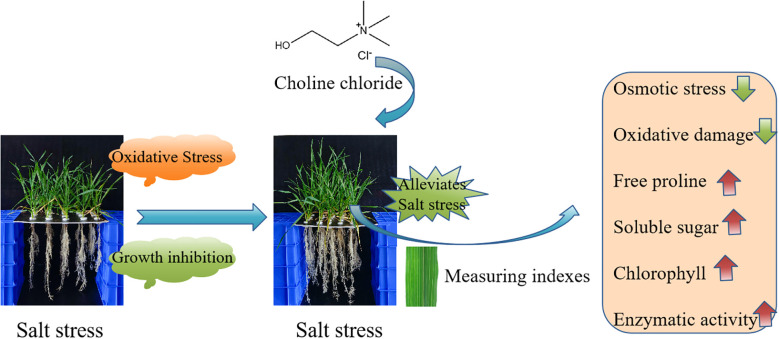

## 1 Introduction

Soil salinization is a global problem that can limit agricultural production and cause food insecurity in affected areas, which in turn restricts social and economic development (Arora et al., 2018; Lin et al., 2017; Lowry et al., 2019; Mora et al., 2020; Singh, 2022). It is well documented that high soil salinity produces numerous negative symptoms in crop plants, including the loss of chlorophyll from the leaves, protoplasm dehydration, impaired transpiration and respiration, the inhibition of cell division and expansion, reduced enzyme activity, membrane dysfunction, ionic imbalances, and altered gene expression, all of which have a deleterious effect on the final yield and quality of the crop (Abbas et al., 2024; Hamouda et al., 2023; Saad-Allah et al., 2022; Arif et al., 2020). In addition, saline conditions can result in high cellular permeability, which leads to water stress, resulting in reduced stomatal opening and therefore reduced rates of photosynthesis, which also limits plant growth and development. Furthermore, salt stress is known to result in an increase in reactive oxygen species (ROS), which causes oxidative damage that can affect nucleic acids, proteins and membrane lipids, and further impair the normal function of plant organelles and cells. It is therefore no surprise that salt stress can result in dramatically reduced growth in crop plants and severely limit their yield (Nazar et al., 2011; Saad-Allah, 2015).

Several approaches have been considered to alleviate such effects, including the breeding of salt-tolerant varieties and soil improvements. However, their application in practice is somewhat limited due to the high requirements of time and effort, as well as the large investment required for implementation. A more convenient solution would be the application of exogenous compounds that could help mitigate the negative effects of salt stress on plants, without the need for such prohibitive interventions. Plant growth regulators (PGRs) are a group of compounds that can promote or inhibit plant growth and development at low concentrations (Shen et al., 2024), and studies have shown that the exogenous application of such compounds can also alleviate the adverse effects of stress in a broad range of agricultural crops (Akram and Ashraf, 2013; Watanabe et al., 2000; Averina et al., 2014; Balestrasse et al., 2010; Matters and Beale, 1994).

Wheat is one of the three major food crops of the world (Shewry, 2009), and as with other crops, it has little defense against salt stress and is highly sensitive to saline conditions, which can severely limit yields. The use of an appropriate PGR would be an extremely practical method to alleviate the adverse effects of salt stress on wheat (Sarika et al., 2023; Zeeshan et al., 2020), and previous studies have demonstrated the effectiveness of such an approach with the application of melatonin (Ke et al., 2018). Another candidate PGR is choline chloride (2-hydroxyethyl trimethylammonium chloride; chemical formula: C_5_H_14_ClNO) a promoter of photosynthesis that has been found to significantly increase yields. Indeed, numerous studies have found that choline chloride can improve resistance to stress, and promote the growth of wheat, rice, cotton and other crops, leading to higher yields (Zhao et al., 2007). Furthermore, several studies have also found that the exogenous application of choline chloride can reduce the effect of salt stress in other crops including cucumber and spinach. The current study evaluated the potential of seed soaking with choline chloride to relieve the symptoms of salt stress in wheat seedlings. The effects on germination and growth were monitored and measured, and biochemical assays used to investigate any physiological changes that might reveal the underlying mechanism for the observed results, and thereby establish whether the application of choline chloride would be a suitable PGR to improve the tolerance of wheat plants to salt stress.

## 2 Materials and methods

### 2.1 Effect of salt stress on the germination rate of wheat seeds under salt stress

The current study adapts an *in vitro* approach described in a previous study to determine the germination rate of wheat seeds in response to saline conditions (Chen et al., 2013). Briefly, full wheat grains (Bainong207; Kindly donated by Professor Ou Xing-qi from Henan Institute of Science and Technology), which were identified by immersion in water and the selection of the ones that sank, were surface-sterilized in 0.5% NaClO for 20 min, washed in distilled water, and soaked in distilled water for 24 h before being placed in fresh petri dishes (30 seeds per dish). Salt solutions of increasing concentration (0, 50, 100, 150, 200, 250, and 300 mmol·L^-1^ NaCl) were added to the petri dishes, which were then incubated at 25°C for 7 days, with the salt solution being replaced on a daily basis. Each treatment consisted of three replicate dishes. The germination status of the seeds was monitored and recorded every day, and the germination rate determined on the 7^th^ day. The data obtained were used to calculate the semi-inhibitory salt concentration for seed germination as described previously (Chen et al., 2013).

### 2.2 Effect of choline chloride seed soaking on wheat seed germination and seedling growth

The seed soaking treatment applied in the current study was adapted from the method of a previous study (Shen et al., 2024). In this case full grain wheat seeds were immersed in increasing concentrations (0, 200, 400, 600, and 800 mg·L^-1^) of choline chloride (Obtained from the Henan Institute of General Chemical Industry Co., LTD.) for a period of 24 h before the excess solution was discarded and the seeds allowed to dry naturally. The germination and growth of the wheat seedlings were then assessed using the *in vivo* method described previously (Han et al., 2023). In this case, seeds of approximately the same size were selected and planted on the surface of culture dish (the diameter is 9cm.) containing vermiculite (30 seeds per pot, each treatment was repeated three times), which were subsequently kept in a growth chamber with a 16 h photoperiod and a day/night temperature of 25°C/20°C. The relative humidity was maintained at 60%, and the daytime light intensity at 400 μmol ·m^-2^ ·s^-1^. The germination status was observed on the 3^rd^ and 7^th^ day, and the data obtained used to calculate the germination potential and germination rate using the following formulae:


$$\text{Germination potential }=\frac{G3}{N}*100$$

$$\text{Germination rate }=\frac{G7}{N}*100$$

Where G_3_ and G_7_ represent the number of germinated seeds per pot on day 3 and day 7, respectively, and N the total number of seeds per pot.

On the 7^th^ day of the experiment 15 plants were randomly selected from each treatment and their growth parameters recorded, including plant height and root length, as well as the fresh weight of their above- and below-ground parts. The corresponding dry weight values were determined after drying in an oven at 85°C.

### 2.3 Effect of choline chloride seed soaking on wheat seed germination and seedling growth under salt stress

Wheat seeds were prepared and treated with various concentrations of choline chloride as described above (1.1 and 1.2, respectively), before being subjected to salt stress according to the protocol from a previous study (Qiu et al., 2014). However, in this case the semi-inhibitory salt concentration (120 mmol·L^-1^ NaCl) from the preliminary experiment (1.1) was used. Briefly, seedlings were established in culture dish containing vermiculite as described above (2.1), before being transferred to hydroponic trays(It is 30cm long, 20cm wide and 8.7cm high) at the two true leaf stages, each treatment was repeated three times, and 20 wheat seedlings were cultured in each hydroponic dish. The seedlings were then returned to the growth chamber and irrigated with Hoagland nutrient solution and the growth chamber with a 16 h photoperiod and a day/night temperature of 25°C/20°C. The relative humidity was maintained at 60%, and the daytime light intensity at 400 μmol ·m^-2^ ·s^-1^. The Hoagland nutrient solution concentration was increased on a daily basis from 1/8 to 1/4 and finally to 1/2. On the 7^th^ day, add 120 mmol ·L^-1^ NaCl, where it remained until the end of the experiment. On the 7^th^ day of NaCl stress treatment, 5 wheat seedlings were randomly selected from each of the treatments, which have been summarized in **Table 1**, and their growth parameters assessed as described above (1.2.1).

**Table 1:** Test treatments.

Treatment	Seed soak	Nutrient solution	Salt stress (mmol·L^-1^ NaCl)
Control	Water	1/2 Hoagland nutrient solution	/
Water + NaCl stress	Water	1/2 Hoagland nutrient solution	120
200 mg·L^-1^CC + NaCl stress	200 mg·L^-1^ choline chloride	1/2 Hoagland nutrient solution	120
400 mg·L^-1^CC + NaCl stress	400 mg·L^-1^ choline chloride	1/2 Hoagland nutrient solution	120
600 mg·L^-1^CC + NaCl stress	600 mg·L^-1^ choline chloride	1/2 Hoagland nutrient solution	120
800 mg·L^-1^CC + NaCl stress	800 mg·L^-1^ choline chloride	1/2 Hoagland nutrient solution	120

### 2.4 Effect of choline chloride seed soaking on the physiology of wheat seedlings under salt stress

Wheat seedlings were established and subjected to salt stress as described above (1.3), and leaf samples collected on the 7^th^ day of the treatment for assessment in the following biochemical assays:

#### 2.4.1 Determination of chlorophyll content

The total photosynthetic pigment content of the leaves was assessed using the method of a previous study (Zhu et al., 2018) in conjunction with a plant chlorophyll kit (Nanjing Jiengcheng Bioengineering Institute). Leaf samples (0.1 g) were collected from each treatment and processed according to the instructions of the kit manufacturer taking care to avoid exposure to light. When the extraction in a 1:2 (v/v) mixture of anhydrous ethanol/acetone was complete, the absorbance of each sample was measured at both 645 nm and 633 nm using a spectrophotometer. The chlorophyll a, chlorophyll b and total chlorophyll content (mg·g^-1^ fresh tissue) were then calculated using the following formulae:


$$Chlorophyll\;a\;content=(12.7\times A_{663}-2.69\times A_{645})\times V_{\text{homogenate}}\times (N/W)/1000$$

$$Chlorophyll\;b\;content=(22.9\times A_{645}-4.68\times A_{663})\times V_{\text{homogenate}}\times (N/W)/1000$$

$$Total\;chlorophyll\;content=(20.21\times A_{645}+8.02\times A_{633})\times V_{\text{homogenate}}\times (N/W)/1000$$

Where V_homogenate_ represents the total volume of leaf homogenate (5 mL); N, the dilution ratio; and W, the mass of the sample assessed (0.1g).

#### 2.4.2 Determination of malondialdehyde content

The malondialdehyde (MDA) content of the wheat leaves was determined using the malondialdehyde test box (Nanjing Jiengcheng Bioengineering Institute). In this case, 0.1 g samples of plant tissue were mechanically homogenized in 10 ml phosphate buffer saline (PBS) (0.1 M) using an ice bath to maintain a low temperature. The cell debris was removed by centrifugation (12000 rpm for 10 min), and the resulting supernatant treated with the kit reagent before the absorbance at 532 nm was measured, and the MDA content (nmol/mg protein) calculated using the following formula:


$$MDA\;content=(A_{532}\;sample-A_{532}\;control)/(A_{532}\;standard-A_{532}\;control)\times C_{standard}/P$$

Where C_standard_ represents the concentration of the standard (10 nmol ·mL^-1^); and P, the total protein content of the homogenate (1 nmol ·mgprot^-1^).

#### 2.4.3 Determination of proline content

The current study utilized the ninhydrin method in conjunction with the proline test box (Nanjing Jiancheng Bioengineering Institute) to determine the proline content of fresh leaf samples (0.1 g), which were homogenized in 10 mL extraction buffer at low temperature. The supernatant was collected by centrifugation (3500 rpm for 10 min) and the appropriate reagent was added before the absorbance at 520 nm was measured, and the proline content (μg ·g^-1^ fresh tissue) calculated using the following formula:


$$\begin{array}{c} \text{Proline content}=(A_{520}\;sample-A_{520}\;control)/(A_{520}\;standard-A_{520}\;control)\times \\ C_{standard}/(W/V_{\text{homogenate}})\times N \end{array}$$

Where C_standard_ represents the concentration of the standard (5 μg/mL); W, the mass of the sample tissue (g); V_homogenate_ the volume of the homogenate (mL); and N, the dilution ratio.

#### 2.4.4 Determination of soluble sugar content

The anthrone colorimetric method was applied using the plant soluble sugar test box (Nanjing Jiancheng Bioengineering Institute) to measure the soluble sugar content of the various leaf samples (0.1 g), which were prepared by mechanical homogenization in 10 ml extraction buffer for 10 min at high temperature in a boiling water bath. After cooling, the supernatant was collected by centrifugation (4000 rpm for 10 min) and diluted 1:10 in distilled water before the kit reagent was added and the absorbance at 620 nm measured. The soluble sugar content (μg ·g^-1^ fresh tissue) was then calculated as follows:


$$\begin{array}{c} Soluble\;sugar\;content=(A_{620}\;sample-A_{620}\;control)/(A_{620}\;standard-A_{620}\;control)\times \\ C_{standard}/(W/V_{homogenate})\times N \end{array}$$

Where C_standard_ represents the concentration of the standard (5 μg ·mL^-1^); W, the mass of the sample (g); V_homogenate_, the volume of the homogenate (mL); and N, the dilution ratio.

#### 2.4.5 Determination of total protein content

The BCA microplate method was applied using a protein quantitative kit (Nanjing Jiancheng Bioengineering Institute) to determine the total protein content of 0.1 g samples of plant tissue according to the instructions of the manufacturer. The sample was homogenized in 10 mL phosphate buffer (0.1 M, pH = 7.4) at low temperature, and the cell debris removed by centrifugation (2500 rpm for 10 min). The resulting supernatant was diluted tenfold with a saline solution, before the appropriate reagents were added, and the absorbance at 562 nm measured in order to calculate the total protein concentration (μg ·mL^-1^ homogenate) using the following formula:


$$\begin{array}{c} \text{Protein concentration}=(A_{562}\;sample-A_{562}\;control)/(A_{562}\;standard-A_{562}\;control)\times \\ C_{standard}\times N \end{array}$$

Where C_standard_ represents the concentration of the standard (524 μg ·mL^-1^); and N, the dilution ratio.

#### 2.4.6 Determination of hydrogen peroxide content

The hydrogen peroxide content of 0.1 g leaf samples was determined using the hydrogen peroxide test box (Nanjing Jiengcheng Bioengineering Institute) according to the instructions of the manufacturer. The sample was homogenized on ice in 10 mL phosphate buffer (0.1 M) and the cell debris removed by centrifugation (12,000 rpm for 10 min). The appropriate reagent was added to the decanted supernatant and the absorbance at 405 nm measured to calculate the hydrogen peroxide content (mmol ·g^-1^ fresh tissue) as follows:


$$\begin{array}{c} H_{2}O_{2}\;content=(A_{405}\;sample-A_{405}\;conrol)/(A_{405}\;standard-A_{405}\;control)\times \\ C_{standard}/(W/V_{homogenate}) \end{array}$$

Where C_standard_ represents the concentration of the standard (163 mmol ·L^-1^); W, the mass of the tissue sample (g); and V_homogenate_, the total volume of the homogenate (mL).

#### 2.4.7 Determination of superoxide dismutase activity

The hydroxylamine method of the superoxide dismutase assay kit (Nanjing Jiengcheng Bioengineering Institute) was used to measure the enzyme activity of 0.1 g tissue samples, which were homogenized in 4 mL phosphate buffer (0.1 M) using an ice bath to maintain low temperature. The cell debris was removed by centrifugation (4000 rpm for 10 min) and the kit reagent added to the resulting supernatant before the absorbance at 550 nm was measured in order to calculate the superoxide dismutase (SOD) activity (U ·g^-1^ fresh tissue) using the following formula:


$$SOD\;activity=(A_{550}\;control-A_{550}\;sample)/A_{550}\;control/50\%\times V_{reaction}/(V_{sample}/C_{sample})$$

Where V_reaction_ represents the volume of the reaction mixture (mL); V_sample_ the volume of the homogenate in the reaction mixture (mL); and C_sample_, the concentration of the homogenate (g ·mL^-1^).

#### 2.4.8 Determination of peroxidase activity

The enzyme activity of cell homogenates was determined using the peroxidase assay kit (Nanjing Jiancheng Bioengineering Institute). Briefly, 0.1g samples of plant tissue were prepared by grinding in 10 mL phosphate buffer (0.1 M, pH = 7.0-7.4) using an ice bath to maintain a low temperature. The cell debris was removed by centrifugation (4000 rpm for 10 min) and the appropriate reagent added to the resulting supernatant. The peroxidase (POD) activity (U·g^-1^ fresh tissue) was then determined from the absorbance value at 420 nm using the following formula:


$$\begin{array}{c} POD\;activity=(A_{420}\;sample-A_{420}\;control)/(12\times d)\\ \times (V_{reaction}/V_{sample})/T/(W/V_{homogenate})\times 1000 \end{array}$$

Where V_reaction_ represents the total volume of the reaction mixture (4 mL); V_sample_ the volume of homogenate in the reaction mixture (0.1 mL); T, the reaction time (min); W, the mass of the tissue sample (g); and V_homogenate_, the total volume of homogenate (10 mL).

#### 2.4.9 Determination of catalase activity

The ammonium molybdate method for determining catalase activity was used by processing the various tissue samples with the catalase assay kit (Nanjing Institute of Biological Engineering) Tissue homogenates were prepared as described above (1.4.8) and the kit reagents added to the resulting supernatant. The absorbance at 405 nm was measured in order to calculate the catalase (CAT) activity (U·g^-1^ fresh tissue) as follows:


$$CAT\;activity=(A_{405}\;control-A_{405}\;sample)\times 271/V_{sample}/T/(W/V_{homogenate})$$

Where, V_sample_ represents the volume of homogenate in the reaction mix; T, the reaction time (min); W, the mass of the tissue sample (g), and V_homogenate_, the total volume of the homogenate (mL).

#### 2.4.10 Determination of ascorbate peroxidase activity

The ascorbate peroxidase test box (Nanjing Jiengcheng Bioengineering Institute) was used to assess the enzyme activity of tissue homogenates that were prepared as described previously (1.4.8), but in this case with centrifugation at 10,000 rpm for 10 min. The appropriate reagent was added to the resulting supernatant and the absorbance at 290 nm measured at two time points during the reaction, namely at 10 s (A_0_) and 130 s (A_1_). The values obtained were used to calculate the ascorbate peroxidase (APX) activity (U/g fresh tissue) as follows:


$$\begin{array}{c} APX\;activity=(\Delta A_{290}\;sample-\Delta A_{290}\;control)/(\epsilon \times d)\times \\ \left(V_{reaction}\times V_{homogenate})/(V_{sample}\times W)/(T\times N\right) \end{array}$$

Where ϵ represents the extinction molar coefficient (2.8 μmol ·mL^-1^cm^-1^); V_reaction_, the total volume of the reaction mixture (mL), V_homogenate_ the total volume of the homogenate (mL); V_sample_, the volume of homogenate in the reaction mixture (mL), W, the mass of the tissue sample (g); T, the reaction time (min); and N, the dilution ratio.

#### 2.4.11 Determination of polyphenol oxidase activity

The polyphenol oxidase test box (Nanjing Jiancheng Bioengineering Institute) was used to assess the enzyme activity of leaf homogenates (1.4.8). In this case, the cell debris was removed by centrifugation (8000 rpm, for 10 min), before the kit reagent was added to the supernatant and the absorbance at 420 nm used to calculate the polyphenol oxidase (PPO) activity (U ·g^-1^ fresh tissue) as follows:


$$\begin{array}{c} PPO\;activity=(A_{420}\;sample-A_{420}\;control)/0.01\times (V_{homogenate}/W)\times \\ (V_{reaction}/V_{sample})/V_{reaction}/T \end{array}$$

Where V_homogenate_ represents the total volume of homogenate (mL); W, the mass of the tissue sample (g); V_reaction_ the total volume of the reaction mixture (mL); V_sample_, the volume of homogenate in the reaction mixture (mL); and T, the reaction time (min).

### 2.5 Statistical analysis

The data obtained from the various experiments were analyzed using SPSS (26.0) software, with significant differences between treatments determined according to Duncan’s new complex range test (*p* ≤ 0.05).

## 3 Results and analysis

### 3.1 Effect of salt stress on wheat seed germination

**Figure 1:**
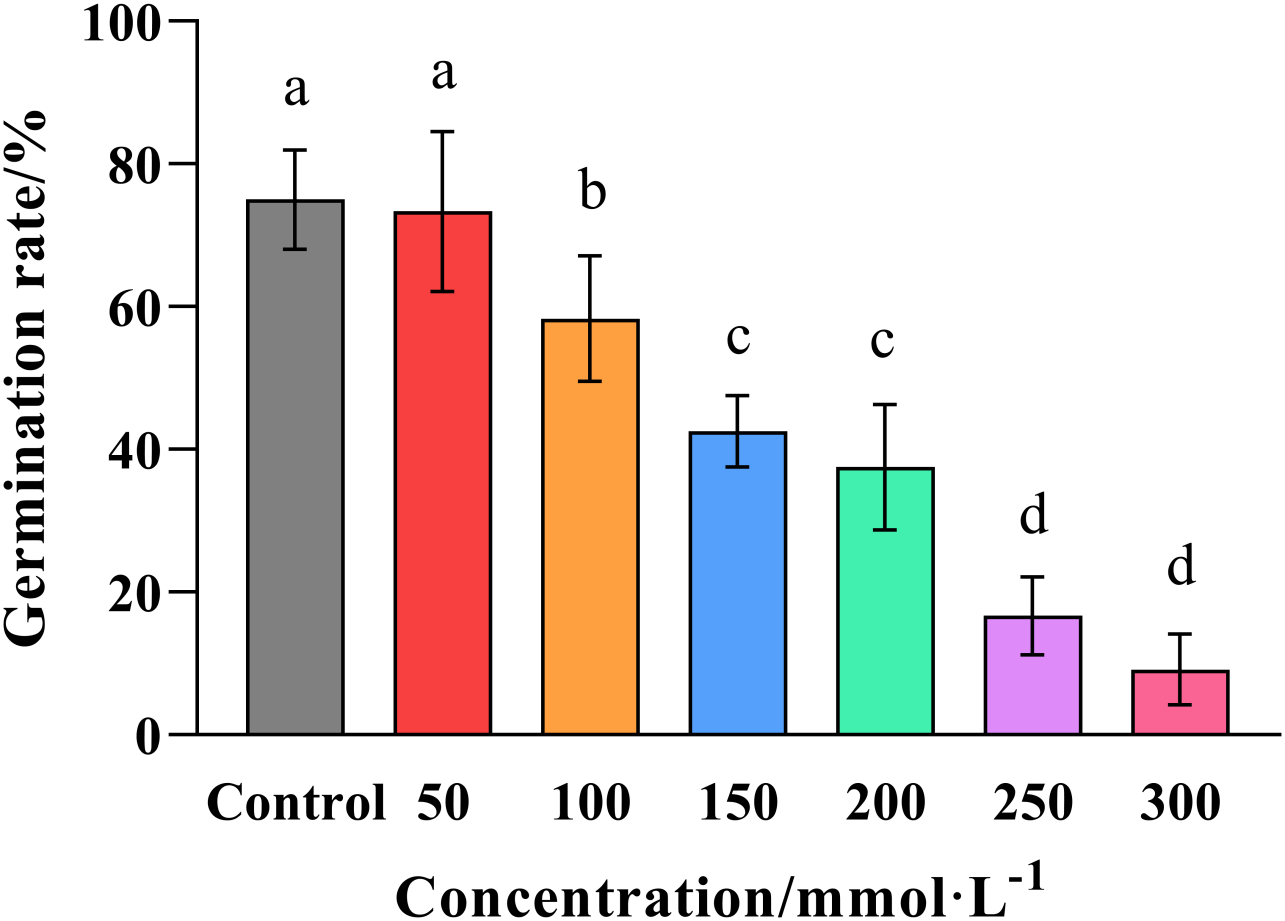
Effect of salt concentration on the germination of wheat seeds. Graphed data are shown as means ± standard errors.

The results of the *in vitro* assays showed that the germination rate of wheat seeds decreased with increasing salt concentration (**Figure 1**). However, the effect only became significant (*p ≤ 0.05*) at a concentration of 100 mmol·L^-1^ NaC1, while the germination rate diminished dramatically at higher concentrations falling to 42.52% at 150 mmol·L^-1^, and just 9.17% at 300 mmol ·L^-1^. Meanwhile, linear regression revealed that the salt concentration required for a 50% reduction in germination was 119.61 mmol·L^-1^, so the rounded figure of 120 mmol·L^-1^ was used to represent the semi-inhibitory dose when we investigating the effects of salt stress in the subsequent experiments.

The germination rate of wheat seeds was determined by submerging them in solutions of increasing salt concentration (0, 50, 100, 150, 200, 250, and 300 mmol ·L^-1^ NaCl), and counting the number of germinated seeds after 7 days of treatment. Bars indicate one standard deviation (SD), while different letters above the columns indicate significant differences (*p* ≤ 0.05) according to Duncan’s new complex range test.

### 3.2 Effect of choline chloride on wheat seed germination and seedling growth

**Figure 2:**
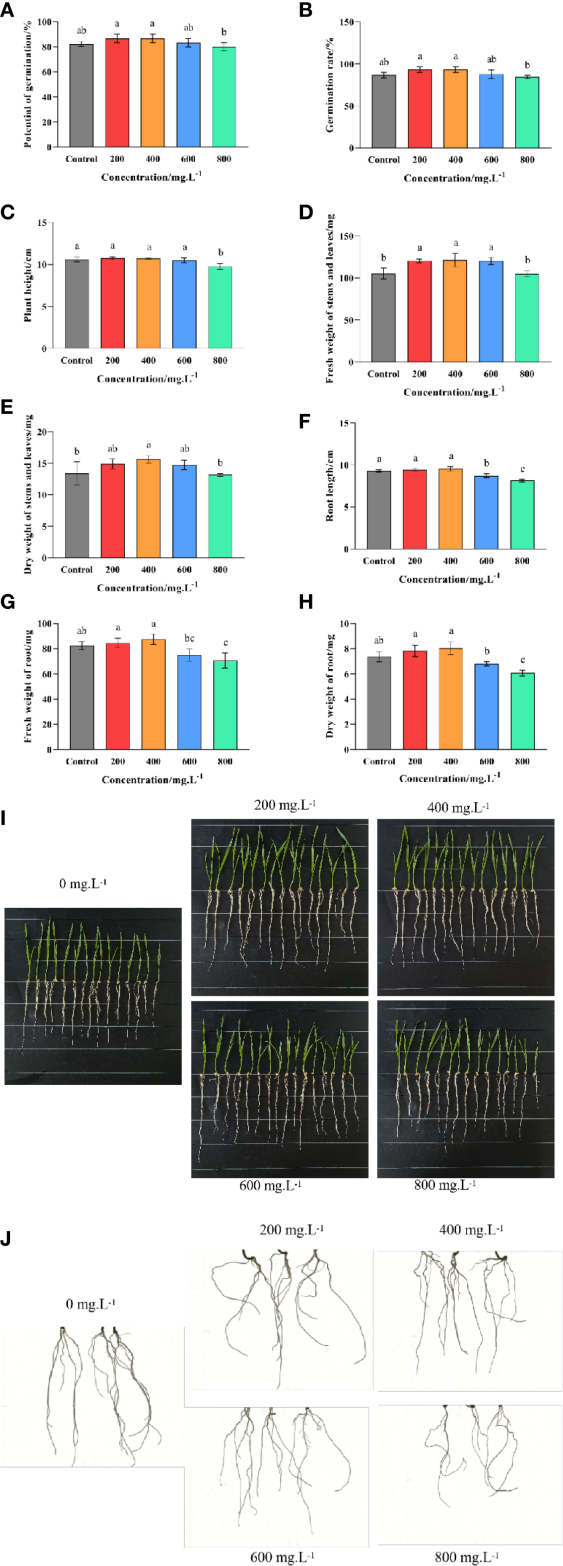
Effect of choline chloride on wheat seed germination and seedling growth. Graphed data are shown as means ± standard errors. The panels below show the effect of different concentrations of choline chloride (200, 400, 600, and 800 mg·L^-1^) on plant height and root length **(I)**, as well as root morphology **(J)**, while the graphs above show the corresponding data regarding seed germination potential **(A)**, observed germination rate **(B)**, plant height **(C)**, root length **(D)**, fresh and dry weight of above-ground tissue **(E, F)**, and the fresh and dry weight of below-ground tissue **(G, H)**. Bars indicate one standard deviation (SD), while different letters above the columns indicate significant differences (*p ≤ 0.05*) according to Duncan’s new complex range test.

Pot experiments revealed that soaking seeds in a choline chloride solution had a significant (*p ≤ 0.05*) effect on the germination and growth of wheat (**Figure 2**). In general, the lower doses (200–400 mg·L^-1^) of choline chloride had a mild stimulatory effect, while the higher doses (600–800 mg·L^-1^) could result in negative effects. For example, both the potential germination and the observed germination rates were found to be significantly (*p ≤* 0.05) increased (5.41% and 7.69%, respectively) at 200 and 400 mg·L^-1^, but unaffected at 600 mg·L^-1^, and significantly (*p ≤ 0.05*) reduced 2.56% at 800 mg·L^-1^ compared to the untreated control (**Figures 2A, B**). Meanwhile, it was found that although low to moderate doses of choline chloride (200–600 mg·L^-1^) had no discernable effect on the height of the wheat seedlings, they did result in an increase in both the fresh and dry weight of the above ground tissue (an increase of 14.51%, 15.30% and 14.29%; and 11.19%, 16.67% and 9.95%, respectively), while in contrast the highest dose (800 mg·L^-1^) resulted in reduced height, but no significant difference in mass (**Figures 2C, E, F**). Similarly, low doses (200–400 mg·L^-1^) of choline chloride had no effect on root length, but resulted in an increase in the below ground biomass (2.59% and 6.19%, and 6.33% and 9.05% for fresh and dry mass, respectively), while higher doses (600–800 mg·L^-1^) caused both a reduction in root length (6.20% and 13.68, respectively) as well as a corresponding reduction in the below ground biomass (9.10% and 14.23%, and 7.69% and 17.65% for fresh and dry mass, respectively (**Figures 2D, G, H**). Taken together these results indicate that the ideal choline chloride dose to promote the growth of wheat lies in the 200–400 mg·L^-1^ range.

### 3.3 Effect of choline chloride on the growth of wheat seedlings experiencing salt stress

**Figure 3:**
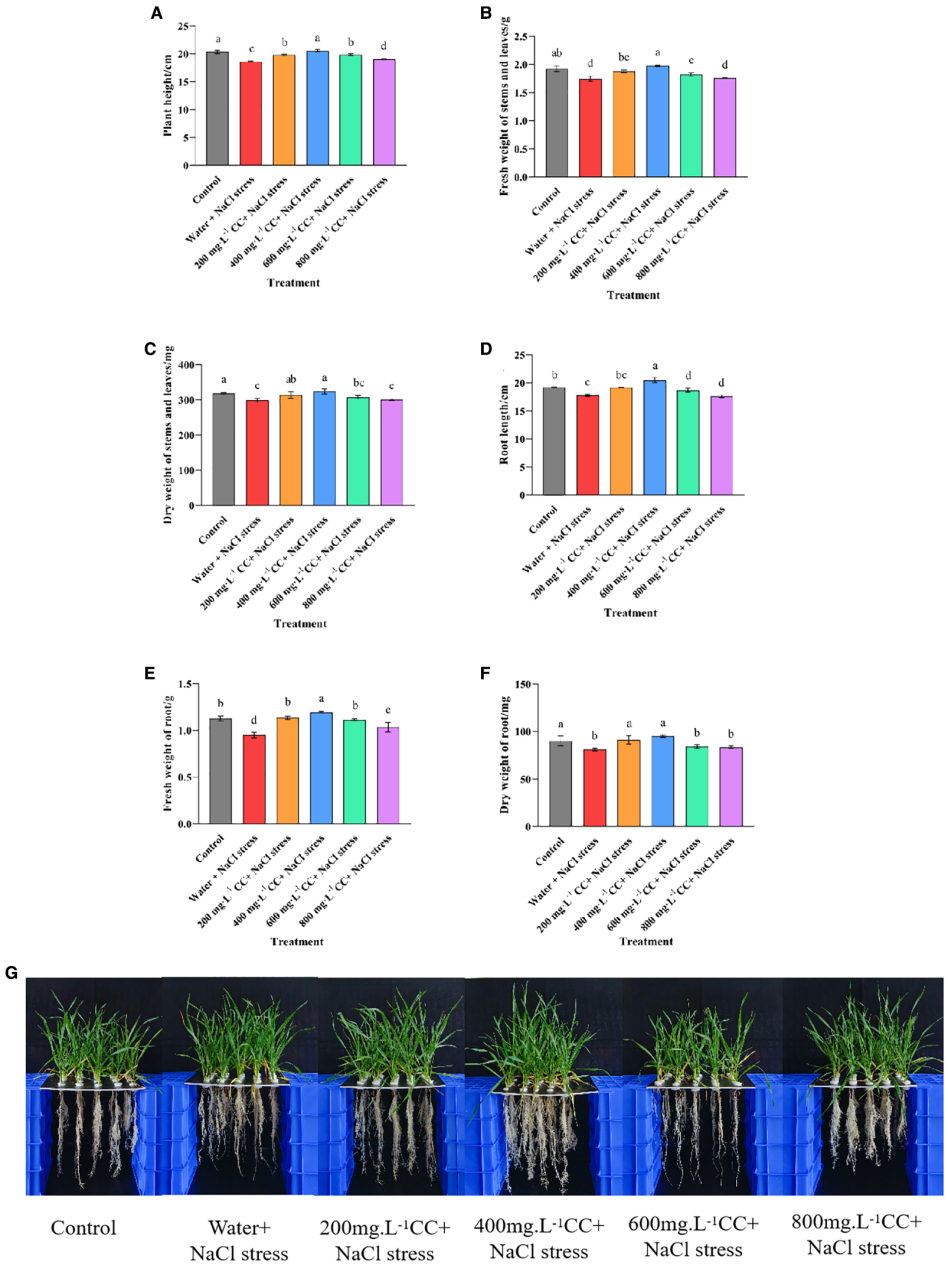
Effect of choline chloride on the growth of wheat seedlings experiencing salt stress. Graphed data are shown as means ± standard errors. The panels below **(G)** show the effect of different concentrations of choline chloride (200, 400, 600, and 800 mg·L^-1^, respectively) on wheat seedlings exposed to salt stress (120 mmol ·L^-1^ NaCl), compared to the untreated negative control (Water and 120 mmol ·L^-1^ NaCl stress), and seedlings not experience salt stress (Control), while the graphs above show the corresponding data for plant height **(A)**, root length **(D)**, fresh and dry weight of above-ground tissues **(B, C)**, and the fresh and dry weight of below-ground tissue **(E, F)**. Bars indicate one standard deviation (SD), while different letters above the columns indicate significant differences (*p ≤ 0.05*) according to Duncan’s new complex range test.

The growth of the wheat seedlings was evaluated after 7 days of exposure to salt stress (120 mmol ·L^-1^ NaCl). The results indicated that seed soaking with choline chloride exhibited great potential to ameliorate the symptoms of salt stress, as all of the choline chloride treatments were found to either significantly (*p ≤ 0.05*) promote growth relative to the negative control (water + 120 mmol ·L^-1^ NaCl stress), or in the case of the highest dose (800 mg·L^-1^) have no significant (*p ≤ 0.05*) deleterious effects. However, there was some degree of variation when the different choline chloride treatments were compared to each other, or to the positive control (Control), which was grown in the absence of the salt stress (**Figure 3**). Again, the lower doses seem to produce greater benefits, with the 400 mg·L^-1^ dose resulting in a significant (*p ≤ 0.05*) increase in root length (6.73%) as well as a general increase in biomass compared to the positive control (Control). This dose was also the most effective in comparison to the negative control (Water and 120 mmol ·L^-1^ NaCl stress), resulting in a significant (*p ≤ 0.05*) increase in both height (10.43%) and root length (15.38%), as well as a corresponding increases in the fresh and dry weight of the above- (13.12%, and 8.23%, respectively), and below-ground (25.66% and 17.26%, respectively) tissues. Taken together these results indicate that treatment with choline chloride not only has the potential to relieve the symptoms of salt stress, but at the correct dose, even to promote growth beyond that of untreated plants not experiencing salt stress.

### 3.4 Effect of choline chloride on the physiology of wheat seedlings experiencing salt stress

#### 3.4.1 Chlorophyll content

**Figure 4:**
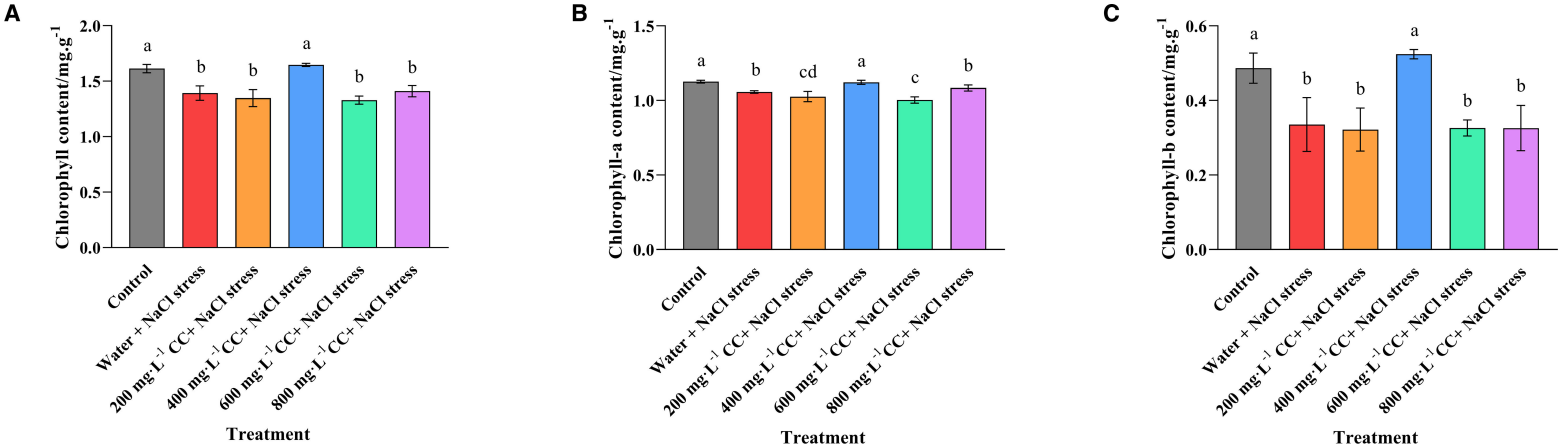
Effect of choline chloride on the chlorophyll content of wheat leaves experiencing salt stress. Graphed data are shown as means ± standard errors. The three graphs show the effect of different concentrations of choline chloride (200, 400, 600, and 800 mg·L^-1^, respectively) on the total chlorophyll **(A)**, chlorophyll a **(B)**, and chlorophyll b **(C)** content of wheat seedlings exposed to salt stress (120 mmol ·L^-1^ NaCl) in comparison to the untreated negative control (Water and 120 mmol ·L^-1^ NaCl stress), and seedlings not experiencing salt stress (Control). Bars indicate one standard deviation (SD), while different letters above the columns indicate significant differences (*p ≤ 0.05*) according to Duncan’s new complex range test.

Comparison of the positive and negative controls, in which untreated seedlings were grown in either the absence (Control) or presence (Water and 120 mmol ·L^-1^ NaCl stress) of the salt stress revealed a significant reduction in both chlorophyll a (6.18%) and chlorophyll b (31.10%) content, as well as a reduction in total chlorophyll (13.14%) content, as a consequence of exposure to the salt stress (**Figure 4**). Furthermore, it was found that treatment with choline chloride (200, 400, 600, and 800 mg·L^-1^) could only provide remediation at the optimal concentration of 400 mg·L^-1^, which restored the chlorophyll levels to that of the positive control (Control), and constituted an increase of 6.21%, 56.36% and 18.32%, for chlorophyll a, chlorophyll b and total chlorophyll, respectively, in comparison to the negative control (Water and 120 mmol ·L^-1^ NaCl stress). Meanwhile, treatment with lower or higher concentrations of choline chloride (200 mg·L^-1^, and 600 mg·L^-1^ and 800 mg·L^-1^ respectively) resulted in no significant change to the chlorophyll levels in comparison to the untreated negative control (Water and 120 mmol ·L^-1^ NaCl stress).

#### 3.4.2 Osmoregulation

**Figure 5:**
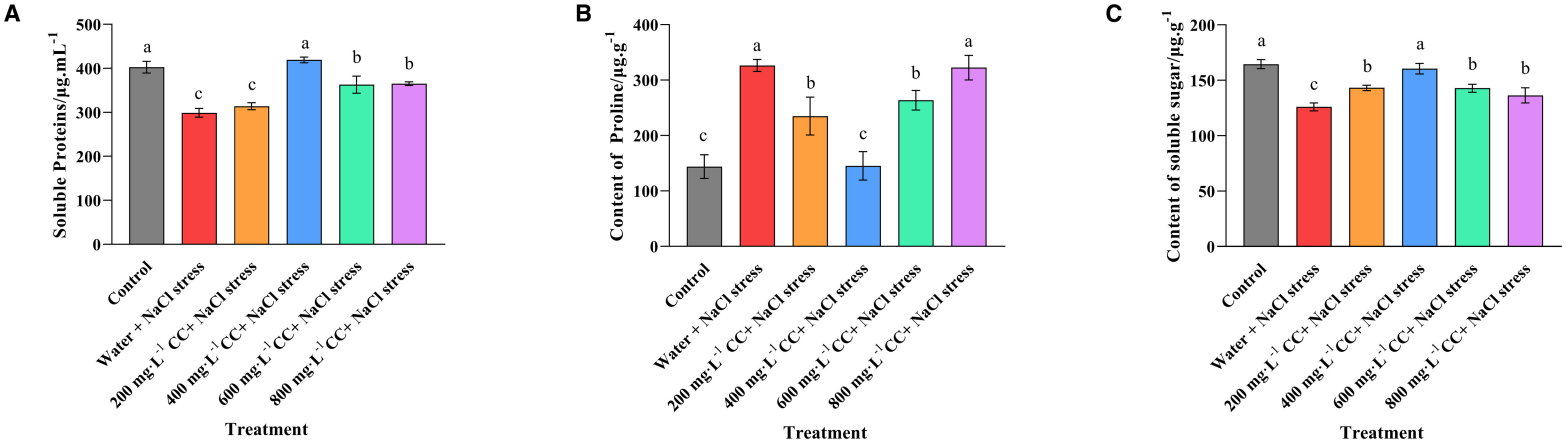
Effect of choline chloride on metabolites associated with osmoregulation in wheat seedlings experiencing salt stress. Graphed data are shown as means ± standard errors. The three graphs show the effect of different concentrations of choline chloride (200, 400, 600, and 800 mg·L^-1^, respectively) on the soluble protein **(A)**, soluble proline **(B)**, and soluble sugar **(C)** content of wheat seedlings exposed to salt stress (120 mmol ·L^-1^ NaCl) in comparison to the untreated negative control (water + 120 mmol ·L^-1^ NaCl stress), and seedlings not experiencing salt stress(Control). Bars indicate one standard deviation (SD), while different letters above the columns indicate significant differences (*p ≤ 0.05*) according to Duncan’s new complex range test.

The levels of soluble protein, proline and sugar were assessed to gauge the effect of salt stress on the osmoregulation of the wheat seedlings (**Figure 5**). The results revealed that the salt stress caused a significant reduction in the protein and sugar content (a 25% and 25% reduction, respectively) of the negative control (Water and 120 mmol ·L^-1^ NaCl stress), but a significant increase in the proline content (125% increase) relative to the positive control (Control), which was not experiencing salt stress. The various choline chloride treatments (200, 400, 600, and 800 mg·L^-1^, respectively) were found to reduce this effect to some degree, but only the 400 mg·L^-1^ treatment was able to restore the concentration of these metabolites to levels that were not significantly (*p ≤ 0.05*) different to those seen in the positive control (Control), which corresponded to a 50% reduction in soluble proline, and an increases in the protein and sugar content (25% and 25%, respectively) in comparison to the untreated negative control (Water and 120 mmol ·L^-1^ NaCl stress).

#### 3.4.3 Oxidative damage

**Figure 6:**
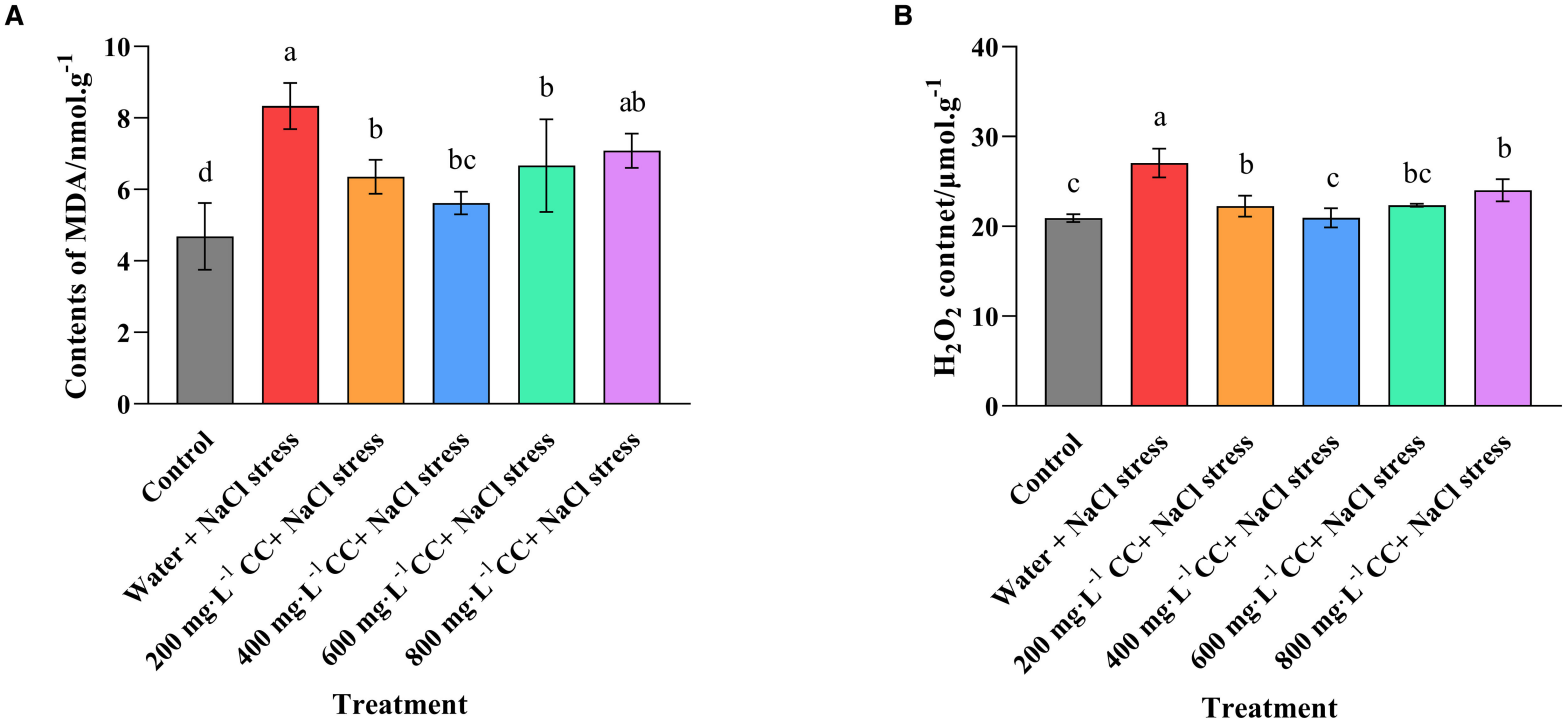
Effect of choline chloride on biomarkers for oxidative stress in wheat seedlings experiencing salt stress. Graphed data are shown as means ± standard errors. The two graphs show the effect of different concentrations of choline chloride (200, 400, 600, and 800 mg·L^-1^; respectively) on the biomarkers for oxidative stress, MDA **(A)**, and H_2_O_2_ **(B)** in wheat seedlings exposed to salt stress (120 mmol ·L^-1^ NaCl) in comparison to the untreated negative control (water + 120 mmol ·L^-1^ NaCl stress), and seedlings not experiencing salt stress (Control). Bars indicate one standard deviation (SD), while different letters above the columns indicate significant differences (*p ≤ 0.05*) according to Duncan’s new complex range test.

An assessment of the biomarkers malondialdehyde (MDA) and hydrogen peroxide (H_2_O_2_) indicated that salt stress caused a significant increase in ROS, when comparing the positive (Control) and negative (water + 120 mmol ·L^-1^ NaCl stress) controls (**Figure 6**). Indeed, the salt stress caused quite a dramatic change, with a 40% increase in the MDA content, and a 25% increase in the H_2_O_2_ content of the leaves. Although treatment with choline chloride was found to ameliorate this accumulation to some degree, there was great variation depending on concentration, and even the most effective treatment (400 mg·L^-1^) was only able to reduce the MDA content to 6 nmol ·g^-1^, which was 50% lower than the negative control (water + 120 mmol ·L^-1^ NaCl stress), but still 50% higher than the positive control (Control). Meanwhile, its H_2_O_2_ content was reduced to levels that were not significantly (*p ≤ 0.05*) different to those of the positive control (Control), while all the other treatments (200, 600, and 800 mg·L^-1^, respectively) still had significantly (*p ≤ 0.05*) elevated levels, even though they were significantly (*p ≤ 0.05*) reduced in comparison to the negative control (water + 120 mmol ·L^-1^ NaCl stress).

#### 3.4.4 Enzyme activity

Having established that salt stress resulted in a significant increase in the biomarkers for oxidative damage, enzyme assays were performed to assess the activity of key enzymes associated with countering the damage caused by ROS. As expected, the activity of all the enzymes was elevated in the negative control (water + 120 mmol ·L^-1^ NaCl stress) compared to the positive control (Control), as the seedlings experiencing salt stress attempted to respond to the associated stress from ROS. However, it was also noted that treatment with choline chloride caused a general increase in enzyme activity, and sometimes to quite a dramatic degree. Again the optimal concentration appeared to be 400 mg·L^-1^, which caused an increase in activities of 119.44%, 44.11%, 15.88%, 12.99%, and 213.45% for APX, SOD, POD, CAT, and PPO, respectively, in comparison to the negative control (water + 120 mmol ·L^-1^ NaCl stress). These results are consistent with the findings that the same treatment (400 mg·L^-1^) could almost completely reduce the accumulation of the biomarkers of oxidative damage (2.4.3), and provide an explanative as to why the growth of the wheat seedlings in this treatment (400 mg·L^-1^) was seemingly completely unaffected by the salt stress (2.3).

## 4 Discussion

Farmers all around the world are facing the challenge of climate change, which is affecting the normal growth of plants and impacting the production of food crops. Meanwhile, the global population is surging, which is increasing both the demand for food, and the need to develop more effective strategies of production. Combating the effects of soil salinity is one such approach, which could both ameliorate the consequences of land salinization as a result of irrigation and other factors, as well as increase the area and potential of marginal farm land, and thereby increase global food security (Wang et al., 2003, 2016; Shi et al., 2002).

Salt stress is one of the major abiotic stresses known to restrict the growth and development of plants, including important crop species. High levels of salinity are known to reduce the osmotic potential of soils, causing ion imbalances that disrupt the physiological processes of plant cells, which in turn inhibits plant growth, reduces the yield and quality of the crop, and in severe cases even leads to plant death. However, there is growing evidence that treatment with exogenous regulatory compounds, commonly known as plant growth regulators (PGR) can increase the chlorophyll content of plant leaves to increase the efficiency of photosynthesis, as well as enhance crop growth as a result of increased tolerance to abiotic stress (Chen and Wang, 2009).

Visible symptoms, such as the yellowing and wilting of leaves, and the stunting of growth, are the most obvious manifestation of saline-alkali damage to plants. However, the process of seed germination itself is particularly sensitive to salt stress, which is known to reduce the rate of germination in a dose-dependent manner. The results of the current study confirmed this phenomenon in wheat, where the rate of germination was observed to diminish dramatically at concentrations in excess of 100 mmol ·L^-1^ NaCl, with the semi-inhibitory concentration being 120 mmol·L^-1^ (**Figure 1**). Similar results have been observed in sweet clover, alfalfa and daisy (Zhanwu et al., 2011; Vu et al., 2015; Tavakkoli et al., 2016), in which the semi-inhibitory concentrations were found to be 120, 150 and 160 mmol ·L^-1^, respectively. The current study also found that the growth of the wheat seedlings was significantly (*p ≤ 0.05*) impaired at 120 mmol·L^-1^, resulting in reduced height, shorter roots and a corresponding reduction in the fresh and dry weight of the above- and below-ground tissues (**Figure 3**). However, seed soaking treatments with the PGR choline chloride was found to ameliorate these symptoms to varying degrees according to the concentration applied, and to actually promote growth with regard to root length, and the fresh and dry weight of the below-ground tissue at the optimal concentration of 400 mg·L^-1^, which also restored the growth of the above-ground tissue to the level observed in the positive control that was not subject to salt stress (**Figure 3**).

Photosynthesis is the key metabolic process responsible for the production of energy in green plants. However, salt and alkali stress are known to block chlorophyll synthesis and cause reduced rates of photosynthesis in most plants (Yang et al., 2011). As might be expected, the results of current study found that salt stress also resulted in reduced chlorophyll content in the leaves of the wheat seedlings. However, treatment with 400 mg·L^-1^ choline chloride was found to completely restore the chlorophyll a, chlorophyll b and total chlorophyll levels to those observed in the control plants not experiencing salt stress. Meanwhile, it was interesting to note that the other concentrations of choline chloride (200, 600, 800 mg·L^-1^) had no positive effects on the chlorophyll content of the wheat leaves, which remained at the same level, or less in the case of chlorophyll a, than those observed in the untreated control exposed to salt stress (**Figure 4**).

Free proline is widely distributed in plant tissues and is recognized as the primary organic regulator of osmotic homeostasis. The accumulation of proline is a defensive response that allows plants to mitigate the effect of salt and alkali stress, and can be used as a signifier that confirms plants are suffering adverse conditions (Butt et al., 2016). The current study found that salt stress significantly increased the accumulation of proline in the cells of the wheat seedlings, and that seed soaking with choline chloride could reverse this process at a concentration of 200 and 600 mg·L^-1^, but particularly at 400 mg·L^-1^, which resulted in proline levels that were not significantly (*p ≤* 0.05) different to those of the control plants that were not experiencing salt stress. It was also noted that choline chloride treatment caused a similar restorative response with regard to the levels of soluble sugar and soluble protein content, with the 400 mg·L^-1^ treatment again resulting in levels of accumulation similar to those of the seedlings not experiencing salt stress. Although the primary role of soluble sugars are to provide energy for the metabolic processes of the cell, and to act as a building block for long chain polymers such as cellulose, they are also important osmoregulatory metabolites (Adams et al., 1992). Meanwhile, soluble protein is known to increases the water retentive capacity of plants cells, and it plays a protective role in the formation of the cell wall and cell membranes (Carrondo, 2003). The observation that choline chloride treatment was able to restore normal levels of soluble proline, sugar and protein is therefore an indication that the treatment was effective in maintaining the water retention capacity of the wheat cells, and thereby helped to mitigate the osmotic stress resulting from high salt concentrations.

**Figure 7:**
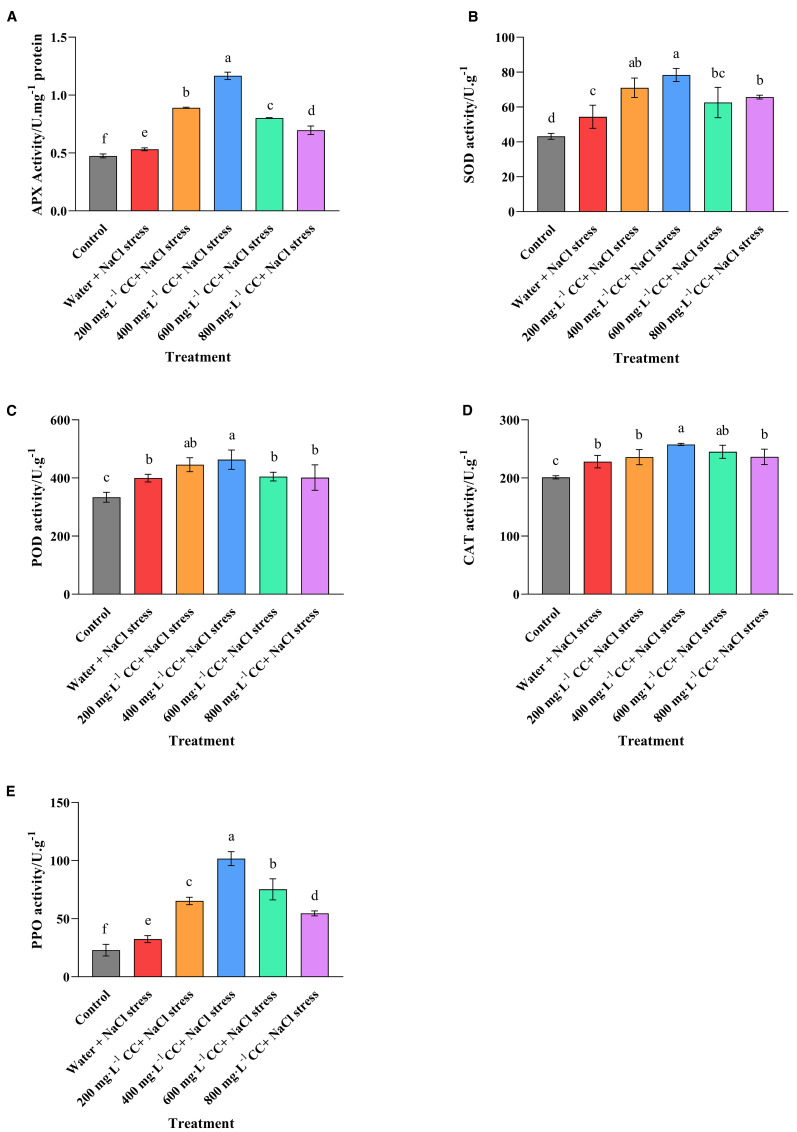
Effect of choline chloride on enzyme activity in wheat seedlings experiencing salt stress. Graphed data are shown as means ± standard errors. The five graphs show the effect of different concentrations of choline chloride (200, 400, 600, and 800 mg·L^-1^; respectively) on the activity (U.g^-1^ fresh tissue) of enzyme associated with the detoxification of ROS, including ascorbate peroxidase **(A)**, superoxide dismutase **(B)**, peroxidase **(C)**, catalase **(D)**, and polyphenol oxidase **(E)**, in wheat seedlings exposed to salt stress (120 mmol·L^-1^ NaCl) in comparison to the untreated negative control (water + 120 mmol·L^-1^ NaCl stress), and seedlings not experiencing salt stress (Control). Bars indicate one standard deviation (SD), while different letters above the columns indicate significant differences (*p ≤ 0.05*) according to Duncan’s new complex range test.

In addition to the well documented effects on chlorophyll and osmoregulation, salt stress is known to cause an accumulation of reactive oxygen species (ROS) that can cause oxidative damage to cell membranes, photosynthetic pigments and important proteins. However, it is also well known that plants have specific defense mechanisms to counter oxidative damage. This includes the production of enzymes such as APX, SOD, CAT, POD, which are essential for neutralizing ROS, as well as PPO, which is also known to play a role in mitigating the effects of oxidative damage and other aspects of abiotic stress (Rangani et al., 2016; Singh and Bhatla, 2016; Scott et al., 1987). The results of the current study confirmed that salt stress did indeed cause oxidative damage to the wheat seedlings as there was a dramatic accumulation of MDA and H_2_O_2_, key biomarkers of oxidative stress. However, treatment with choline chloride was seen to mitigate this process, with all of the treatments resulting in a significant (*p ≤ 0.05*) reduction in the accumulation of these biomarkers (**Figure 6**). The most effective concentration was again 400 mg·L^-1^, which returned H_2_O_2_ to the levels observed in the control seedlings not experiencing salt stress. Enzyme assays revealed the likely cause of these effects, as the activity of all the protective enzymes were found to be significantly increased as a result of the choline chloride treatment (**Figure 7**). Despite this dramatic effect, it was noted that even treatment with 400 mg·L^-1^ choline chloride was unable to completely counter the oxidative damage resulting from the salt stress, as the MDA levels still remained elevated, though they were much lower than those of the untreated negative control. Nonetheless, it seems likely that the protective effect resulting from the increased enzyme activity was the primary factor in allowing the wheat seedlings treated with choline chloride to tolerate salt stress, as it not only reduced the oxidative damage *per se*, but as a consequence, allowed the cells to maintain sufficient chlorophyll levels to sustain normal growth and development.

## 5 Conclusions

The results of the current study confirmed that salt stress hindered the normal growth and development of wheat seedlings and caused significant physiological changes, including reduced chlorophyll levels and increased levels of key biomarkers for osmotic and oxidative stress. However, seed soaking with choline chloride was able to mitigate these effects to varying degrees depending on the concentration. The optimal concentration appeared to be 400 mg·L^-1^, which not only restored most of these biomarkers to the levels observed in seedlings not experiencing salt stress, but also resulted in equivalent growth of above-ground tissues, and an enhancement the growth of the root system. The most likely explanation for these remarkable results was the increased enzyme activity observed in the choline treatment, which not only reduced oxidative damage *per se*, but as a consequence, allowed the cells to maintain sufficient chlorophyll levels to sustain normal growth and development. Although these results indicate that choline chloride treatment could provide a practical solution to the problems associated with soil salinity, further studies are required to confirm not only that the effects persist to maturity and can sustain a productive harvest, but also to determine whether such results can be replicated under field conditions.

## Data Availability

The original contributions presented in the study are included in the article/supplementary material. Further inquiries can be directed to the corresponding authors.
